# Diseases caused by floods with a spotlight on the present situation of unprecedented floods in Pakistan: a short communication

**DOI:** 10.1097/MS9.0000000000000404

**Published:** 2023-04-24

**Authors:** Aroma Naeem, Zaofashan Zaheer, Shehroze Tabassum, Abubakar Nazir, Farhan Naeem

**Affiliations:** King Edward Medical University, Lahore, Pakistan

**Keywords:** floods, infectious diseases, public health

## Abstract

Few natural calamities surpass floods in their destructive capabilities. The recent floods in Pakistan were declared the world’s deadliest since the South Asian floods of 2020 and were, by far, the most destructive floods in the country’s history. They have resulted in significant loss of life and property and have served as the harbingers of cutaneous and noncutaneous diseases. These floods have dealt a critical blow to the country’s already struggling healthcare system, which lacks resources for the prompt mobilization of medical personnel and resources to the flood-hit areas. Having lost their houses, the flood victims are wholly exposed to the elements. Lack of access to a clean water supply has predisposed them to a myriad of diseases. We believe that the consolidation of efforts by the national and international community will put an end to the plight of these flood victims. Our article highlights the significant diseases associated with floods, the challenges faced by the flood victims, and recommendations on how their situation can be improved.

## Introduction

Floods can be defined as an overflow of a large amount of water beyond its normal limits that submerges land after geophysical disasters like heavy rain. Floods represent 40% of the total natural calamities across the globe, making them one of the most significant catastrophes worldwide. Floods can result in intermediate, midterm, and long-term health-related consequences. The immediate health effects caused by floods include acute asthma, respiratory infections, skin rashes, contact dermatitis and clusters, outbreaks of gastroenteritis, and an increase in waterborne diseases^1^. The midterm consequences of floods include infection of wounds due to contaminated water, altered mental health, the spread of communicable diseases, and starvation due to a lack of sufficient food sources^2^. Long-term effects include exacerbation of chronic diseases; poverty-associated diseases like malnutrition are the potential legacy^3^. According to the United Nations High Commissioner for Refugees (UNHCR), more than 90 000 patients seek treatment every day due to recent flood havoc in Pakistan. Furthermore, 3.4 million children need adequate and prompt lifesaving facilities^4^. Five hundred eighty-eight confirmed cases and 10 604 suspected cases of malaria have been reported. The diarrhea outbreak has reached an alarming level with 17 977 cases. By September 2022, skin disease cases inclined to more than 20 064^4^. Cutaneous diseases, along with widespread noncutaneous infectious diseases, have caused massive deaths. According to the recent reports of the United Nations (UN), in October, 1700 people were killed and more than 12 000 got injured during floods in Pakistan^5^.

## Cutaneous diseases caused by floods

### Bacterial and fungal infections

Cellulitis is a common disease among flood sufferers; onset is reported 3–4 days after exposure to floodwater and is seen to persist for up to 3 weeks^6^. *Aeromonas* is a common pathogenic cause of skin infections in patients who come into contact with floodwater^7^. People residing in regions of low immunization coverage are prone to *Clostridium tetani* infection because of contaminated wounds. Melioidosis is a common complication seen in endemic regions due to exposure to contaminated water containing *Burkholderia pseudomallei.* Incidence is seen to rise after heavy floods. Clinical manifestations of Melioidosis include fulminant sepsis, bacteremia, and abscess. Patients with diabetes mellitus, excessive alcohol consumption, and corticosteroid use are more prone to it^8^. Nontuberculous Mycobacterial species are also famous infectious pathogens after floodwater exposure^9^. Fungal infections are also evident among flood-affected individuals^10^. Mucormycosis due to *Rhizopus*, *Mucor*, and *Rhizomucor* are difficult to differentiate because all of these usually present as necrotizing fasciitis and have high mortality rates if not treated properly. The most common site for superficial fungal infection of the skin is the foot due to increased exposure to contaminated water. The clinical manifestation of fungal infection includes erythematous skin maceration and hyperkeratotic papules on the plantar and lateral surface of the foot. Interdigital web spaces of the foot are specifically a common site for fungal infections and are the most common pattern for *tinea pedis* seen in flood victims^11^.

### Traumatic skin diseases

Traumatic skin diseases occur during restoration activities after the flood. Presenting features usually include lacerations, cuts, and penetrating wounds on hands and feet that may lead to secondary bacterial infections. *Staphylococcus aureus* and *Streptococcus pyogenes* are notorious pathogens that get introduced via traumatic wounds.

### Irritant contact dermatitis

Contact dermatitis is a common occurrence due to exposure to chemicals and irritants mixed with floodwater that leads to disruption of the permeability barrier, ultimately resulting in damage to keratinocytes and the release of inflammatory mediators. It usually occurs on the hand and foot and is characterized by erythematous patches; symptoms include burning and stinging sensations along with soreness^12^.

### Insects bite reaction

**Figure 1:**
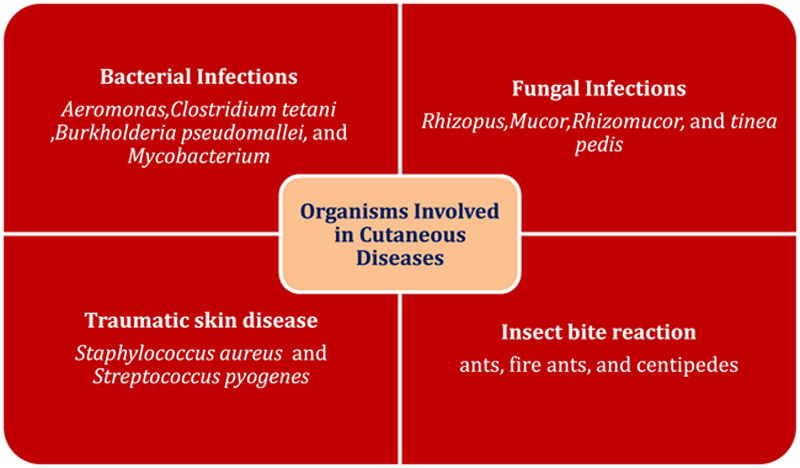
Illustrating organisms involved in cutaneous diseases caused by floods.

The stagnant water provides an optimal breeding environment for mosquitoes. It also causes a surge in the number of insects, including ants, fire ants, and centipedes. The frequency of insect bite reactions is usually high in regions experiencing prolonged floods. Mosquito bite that presents as bullae, vesicles, ecchymosis, and local inflammatory reaction, also known as Skeeter syndrome, is a common occurrence. Vesiculopustular lesions caused by red ants are a common presentation and remain for days to weeks due to the toxic venom. Immediate reactions include wheals and flares followed by a super vesical formation that is edematous in nature^12^. Centipedes are common after floods and can possibly introduce their venom, which causes local inflammatory reactions like the ones caused by bee and wasp stings, a renowned example of hypersensitivity reactions. Common manifestations are painful erythematous swelling patches, bullae formation, and severe itching^13^ (Fig. 1).

## Noncutaneous infectious diseases caused by floods

Outbreaks of waterborne diseases like diarrhea, typhoid, malaria, dengue, and hepatitis A are common. The primary reason is the inadequate availability of purified water and improper sewage system during floods. Transmission of gastrointestinal pathogens via contaminated water during floods is a contributing factor.

### Malaria

Transmission of *Plasmodium falciparum* and *Plasmodium vivax* increases exponentially during monsoon season. Recent floods have played an evident role in malaria epidemics in Pakistan. More than 44 000 new cases have been reported during the last week of September 2022^14^.

### Dengue

From 1st January 2022 till 27th September 2022, confirmed cases of dengue in Pakistan are reported to be 25 932 in number including 62 deaths (case fatality rate is 0.25%). Out of the total number of confirmed cases of dengue, 74% were reported only in the month of September. Heavy floods in the mid of June have markedly contributed to this surge of dengue in Pakistan^15^.

### Respiratory tract infections

Displaced and affected populations with loss of shelter, contaminated drinking water, and increased physical exposure to polluted water are more likely to develop communicable diseases like tuberculosis^16^. Other respiratory infections like pneumonia, common cold, asthma, and allergic bronchitis are also frequent findings among flood victims of Pakistan.

### Hepatitis and diarrhea

**Figure 2:**
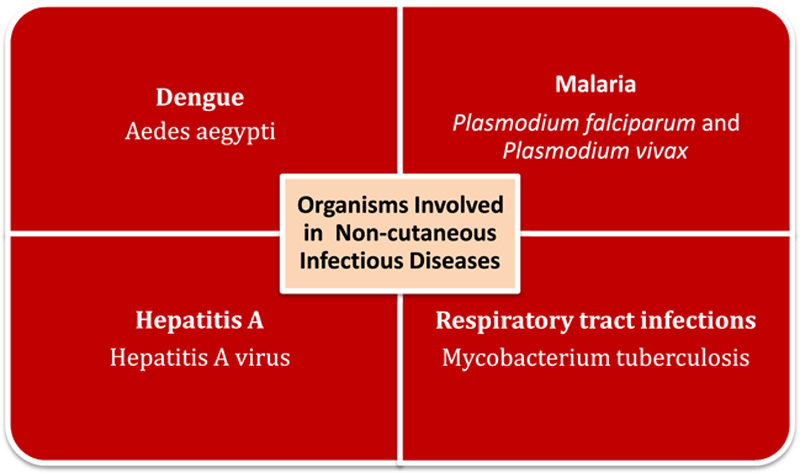
Illustrating organisms involved in infectious diseases caused by floods.

Floodwater is usually contaminated with animal and human wastes, thus containing pathogens transmitted via the fecal–oral route. This poses a threat to the spread of diarrhea, dysentery, and viral hepatitis. In Sindh Province, the number of cases reported merely due to ongoing floods includes over 137 000 cases of diarrhea and more than 10 000 cases of dysentery. Increased incidence of Hepatitis A has also been recorded after floods due to contaminated water in Pakistan^17^ (Fig. 2).

## What are the challenges faced by Pakistan?

**Figure 3:**
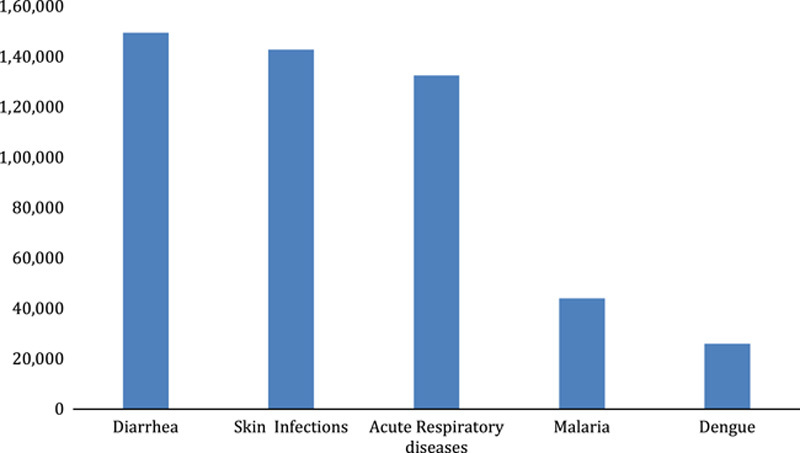
Illustrating the number of cases of diseases caused by floods.

The recent floods in Pakistan add up to the humanitarian crisis, the devastating effects of which far exceed the flood that ravaged the country back in 2010. Pakistan is a developing country, and the disparity that exists between access to healthcare services in rural and urban areas under routine conditions is no secret. This gap has been exaggerated by the recent and relentless flooding, and the country’s healthcare system has been flung into the midst of numerous challenges. Cumulative country-wide deaths and injuries add up to 1606 and 12 863, respectively. Damages to the infrastructure amount to 13 074 in terms of roads and 392 in terms of bridges. A staggering 2 016 025 houses have been destroyed, and according to the Ministry of National Health Services, Regulations and Coordination (MNHSHC), more than 161 000 people have been displaced to relief camps, meaning a huge chunk of the country’s population is currently seeking shelter in temporary camps set up by the government^15,18^. This destruction of infrastructure has severely limited the prompt dispatch of medical personnel and essential medication kits to the flood-affected areas, often leaving on-site medical teams with no choice but to swim to be able to reach the flood victims. This has left the government paralyzed in terms of prompt dispatch of medical personnel and services to the flood-affected. Basic Health Units (BHUs) and rural health camps are being ravaged by floods^19^. Despite the improvements in access to sanitation over the years, 7% of the Pakistani population still practices open defecation^20^. This practice has resulted in the contamination of food and water supplies; and precipitated outbreaks of diseases that spread predominantly via the fecal–oral route. Moreover, the stagnant water continues to serve as breeding grounds for mosquitoes. Outbreaks of diseases like acute watery diarrhea, coronavirus disease 2019 (COVID-19), malaria, and dengue have occurred in camps occupied by flood victims. There have been 149 551 documented cases of diarrhea, 142 739 skin infections, and 132 485 cases of acute respiratory disease. The numbers continue to climb owing to the impossibility of laboratory testing for the diagnosis of these disorders and their subsequent treatment. Data provided by the government show a staggering 600 000 cases of various diseases^21,22^ (Fig. 3).

According to the United Nations Children’s Fund (UNICEF), all the aforementioned conditions have a greater impact on children than on adults. Routine life for these children has come to a halt for the foreseeable future. According to the latest available data, the floods have adversely impacted the lives of over 16 million children, having claimed the lives of as many as 528^23,24^. The flooding and tremendous loss of life have resulted in corpses strewn about on the roads or floating in floodwater. This raises concerns regarding additional loss of life resulting from the spread of pathogens from the cadavers, which typically include bloodborne viruses like HIV and hepatitis; enteric bacteria like salmonella, shigella, and vibrio cholera; viruses like rotavirus, norovirus, hepatitis A virus, and enteric adenovirus; parasites like *Giardia* and *Cryptosporidium*; and airborne agents like *Mycobacterium tuberculosis* ^25^. The emergency departments of most public hospitals in Pakistan struggle to manage patient influx on a daily basis; hospital preparedness in the event of a natural disaster remains a mere pipe dream^26,27^. The hospitals in the hardest hit rural areas have no systems in place for crisis management in emergency situations like safeguarding the hospital staff and timely transport of patients to safety. In addition to the tremendous loss of life and numerous health hazards, the floods have also accentuated the economic and political instability in the country. The destruction caused by flooding constitutes an economic loss of 12.5 billion dollars, and by the end of the year, inflation is predicted to rise to 30%^28^.

## What are the future recommendations at hand?

The quality of drinking water in flood relief camps ought to be promptly assessed to contain the spread of waterborne diseases. People should be warned against using contaminated water and purification techniques like boiling and chemical disinfection should be implemented in emergency situations^29^. Safeguarding vulnerable groups like flood-affected children should be made an utmost priority. Polio vaccinators should be capacitated and special efforts should be directed toward vaccinating children in remote and far-flung flood-affected areas. It is not advisable to rush the disposal of dead bodies in natural disasters because they do not serve as vectors for disease transmission unless communicable diseases and nonphysical injuries from natural disasters are the cause of death. Rather, they should be stored temporarily and personal belongings for later identification should be preserved^30,31^. A greater portion of the budget should be allocated to healthcare. Disaster preparedness training should be made an integral part of the curricula for all healthcare workers. International relief efforts will be crucial in lessening the strain on the already weakened economy of Pakistan. The continued influx of national and international aid will both expedite the recovery process and ensure that the humanitarian response, much needed in these trying times, remains fully funded^32^.

## Ethical approval

Not required.

## Consent

Not applicable.

## Source of funding

None.

## Author contribution

A.N.: conceptualization; A.N., Z.Z., S.T., A.N., and F.N.: writing; A.N. and S.T.: review with critical comments; S.T.: editing.

## Conflicts of interest disclosure

None declared by the authors.

## Research registration unique identifying number (UIN)

1. Name of the registry: not applicable.
2. Unique identifying number or registration ID: not applicable.
3. Hyperlink to your specific registration (must be publicly accessible and will be checked): not applicable.

## Guarantor

Shehroze Tabassum.

## Provenance and peer review

Not commissioned, externally peer-reviewed.
